# Analysis of outcomes in resected early-stage NSCLC with rare targetable driver mutations

**DOI:** 10.1177/17588359241308466

**Published:** 2024-12-23

**Authors:** Nadia Ghazali, Jamie Feng, Katrina Hueniken, Khaleeq Khan, Karmugi Balaratnam, Thomas K. Waddell, Kazuhiro Yasufuku, Andrew Pierre, Laura Donahoe, Elliot Wakeam, Marcelo Cypel, Jonathan Yeung, Shaf Keshavjee, Marc de Perrot, Natasha B. Leighl, Geoffrey Liu, Penelope A. Bradbury, Adrian Sacher, Lawson Eng, Tracy Stockley, Ming Sound Tsao, Frances A. Shepherd

**Affiliations:** Division of Medical Oncology and Hematology, Princess Margaret Cancer Centre (PMCC), University Health Network (UHN), Toronto, ON, Canada; University of Toronto, Toronto, ON, Canada; Division of Medical Oncology and Hematology, Princess Margaret Cancer Centre (PMCC), University Health Network (UHN), Toronto, ON, Canada; University of Toronto, Toronto, ON, Canada; Department of Biostatistics, PMCC, UHN, Toronto, ON, Canada; Division of Medical Oncology and Hematology, Princess Margaret Cancer Centre (PMCC), University Health Network (UHN), Toronto ON, Canada; Division of Medical Oncology and Hematology, Princess Margaret Cancer Centre (PMCC), University Health Network (UHN), Toronto ON, Canada; Division of Thoracic Surgery, UHN, Toronto, ON, Canada; University of Toronto, Toronto, ON, Canada; Division of Thoracic Surgery, UHN, Toronto, ON, Canada; University of Toronto, Toronto, ON, Canada; Division of Thoracic Surgery, UHN, Toronto, ON, Canada; University of Toronto, Toronto, ON, Canada; Division of Thoracic Surgery, UHN, Toronto, ON, Canada; University of Toronto, Toronto, ON, Canada; Division of Thoracic Surgery, UHN, Toronto, ON, Canada; University of Toronto, Toronto, ON, Canada; Division of Thoracic Surgery, UHN, Toronto, ON, Canada; University of Toronto, Toronto, ON, Canada; Division of Thoracic Surgery, UHN, Toronto, ON, Canada; University of Toronto, Toronto, ON, Canada; Division of Thoracic Surgery, UHN, Toronto, ON, Canada; University of Toronto, Toronto, ON, Canada; Division of Thoracic Surgery, UHN, Toronto, ON, Canada; University of Toronto, Toronto, ON, Canada; Division of Medical Oncology and Hematology, Princess Margaret Cancer Centre (PMCC), University Health Network (UHN), Toronto, ON, Canada; University of Toronto, Toronto, ON, Canada; Division of Medical Oncology and Hematology, Princess Margaret Cancer Centre (PMCC), University Health Network (UHN), Toronto, ON, Canada; University of Toronto, Toronto, ON, Canada; Division of Medical Oncology and Hematology, Princess Margaret Cancer Centre (PMCC), University Health Network (UHN), Toronto, ON, Canada; University of Toronto, Toronto, ON, Canada; Division of Medical Oncology and Hematology, Princess Margaret Cancer Centre (PMCC), University Health Network (UHN), Toronto, ON, Canada; University of Toronto, Toronto, ON, Canada; Division of Medical Oncology and Hematology, Princess Margaret Cancer Centre (PMCC), University Health Network (UHN), Toronto, ON, Canada; University of Toronto, Toronto, ON, Canada; Laboratory Medicine Program, UHN, Toronto, ON, Canada; Department of Laboratory Medicine and Pathobiology, University of Toronto, Toronto, ON, Canada; Division of Medical Oncology and Hematology, Princess Margaret Cancer Centre (PMCC), University Health Network (UHN), 700 University Avenue, 7-812, Toronto, ON M5G 2M9, Canada; University of Toronto, Toronto, ON, Canada; Division of Medical Oncology and Hematology, Princess Margaret Cancer Centre (PMCC), University Health Network (UHN), 700 University Avenue, 7-812, Toronto, ON M5G 2M9, Canada; University of Toronto, Toronto, ON, Canada

**Keywords:** actionable genomic alteration, early-stage, non-small-cell lung cancer, rare driver mutation, surgery

## Abstract

**Background:**

Given advancements in adjuvant treatments for non-small-cell lung cancer (NSCLC) with epidermal growth factor receptor (EGFR) and anaplastic lymphoma kinase (ALK)-targeted therapies, it is important to consider postoperative targeted therapies for other early-stage oncogene-addicted NSCLC. Exploring baseline outcomes for early-stage NSCLC with these rare mutations is crucial.

**Objectives:**

This study aims to assess relapse-free survival (RFS) and overall survival (OS) in patients with resected early-stage NSCLC with rare targetable driver mutations.

**Methods:**

This retrospective single-center study identified stage I–III NSCLC patients with rare targetable mutations who underwent curative surgery. Tissue-based molecular profiling identified mutations in *KRAS*G12C, *EGFR* Exon20, Erb-B2 receptor tyrosine kinase 2 (*ERBB2*), *ALK*, *ROS1*, B-Raf proto-oncogene (*BRAF*) V600E, mesenchymal–epithelial transition factor (*MET*) exon14 skipping, and rearranged during transfection (*RET*). Baseline patient and tumor characteristics, mutation subtype, and *TP53* co-mutation were correlated with RFS and OS using Cox regression. The *KRAS*G12C cohort was used as the reference for survival comparisons.

**Results:**

Among 225 patients, mutations included the following: *KRAS*G12C (*n* = 101, 45%), *MET* exon 14 skipping (*n* = 26, 12%), *EGFR* Exon 20 (*n* = 25, 11%), *ERBB2* (*n* = 25, 11%), *ALK* fusion (*n* = 16, 7%), *ROS1* fusion (*n* = 14, 6%), *BRAF* V600E mutation (*n* = 13, 6%), and *RET* fusion (*n* = 5, 2%). Five-year survival probabilities were 76% for stage I, 60% for stage II, and 58% for stage III. RFS was shorter across most mutation subgroups compared to *KRAS*G12C, with *ROS1* mutations showing significantly poorer RFS (HR 2.70, *p* = 0.019). By contrast, all mutation subgroups were associated with better OS than *KRAS*G12C. The incidence of brain metastasis was highest in *ERBB2* (22% at 5 years). TP53 co-mutation was associated with significantly worse OS (HR 2.35, *p* = 0.008).

**Conclusion:**

While RFS was poorer for most mutations compared to *KRAS*G12C, OS generally was better, suggesting a potential role for postoperative targeted therapies. These findings warrant further investigation through prospective studies and clinical trials to optimize adjuvant treatment strategies for patients with early-stage NSCLC harboring rare driver mutations.

## Plain language summary

Outcomes of patients with resected early-stage lung cancer with rare targetable driver mutations

Early-stage non-small cell lung cancer (NSCLC) is often treated with surgery and sometimes chemotherapy after surgery. Recent advancement with discovery of mutations that drive cancer has led to improvement of outcomes in NSCLC with targeted therapies. Certain mutations in NSCLC are rare and how these mutations impact outcomes after surgery in early-stage NSCLC is less clear. Our study focused on patients with early-stage NSCLC who had surgery and specific rare mutations including EGFR Exon20 insertion, KRAS G12C, ALK, ROS1, BRAF, NTRK1/2/3, MET exon 14 skipping, RET, and ERBB2 mutations. We want to understand how presence of these mutations affects the chance of cancer returning (relapse-free survival) and overall survival after surgery. We found that most mutations had higher risk of cancer returning (relapse-free survival) compared to patients with KRAS mutation and ROS1 had highest risk of cancer returning in our study. However, for overall survival all mutations were linked to better overall survival than KRAS mutation. ERBB2 mutation had highest risk to develop brain metastasis when the cancer returns. Those with additional TP53 mutation, another genetic change had worse overall survival outcome. These results provide more evidence regarding the outcome of these patients with rare mutations in early-stage NSCLC. There might be a benefit of using targeted therapies early in the diagnosis of early-stage NSCLC. We hope our results help to inform potential studies in the future to show benefit of using targeted therapies after surgery in early-stage NSCLC.

## Introduction

Lung cancer is one of the leading causes of cancer death worldwide.^1^ Non-small-cell lung cancer (NSCLC) is the most common subtype of lung cancer, but only 25%–30% of patients with NSCLC present with potentially curable, surgically resectable disease.^2^ Furthermore, the percentage of patients who recur or die following surgery for early-stage NSCLC remains high (ranging from 45% with stage IB to 75% with stage III), even with the use of postoperative chemotherapy.^3^ This highlights the need for better adjuvant therapies to improve outcomes.

The treatment landscape of NSCLC has evolved recently with advances in molecular profiling especially next-generation sequencing leading to the discovery of treatable driver oncogenes and personalized medicine based on individual tumor genetic changes.^4^ The identification of actionable genomic alterations, such as epidermal growth factor receptor (*EGFR*) mutations and anaplastic lymphoma kinase (*ALK*) fusions, has led to the development and approval of targeted therapies that inhibit these oncogenic pathways. These targeted therapies have become the standard of care in the first-line treatment of advanced NSCLC harboring these genomic alterations, resulting in improved outcomes compared to traditional chemotherapy. The National Comprehensive Cancer Network (NCCN) recommends complete molecular profiling to identify mutations in *EGFR*, Kirsten rat sarcoma viral oncogene homolog (*KRAS*), *ALK*, c-ros Oncogene 1 (*ROS1*), B-Raf proto-oncogene (*BRAF*), neurotrophic tyrosine receptor kinase 1/2/3 (*NTRK1/2/3*), mesenchymal–epithelial transition factor (*MET*), rearranged during transfection (*RET*), and Erb-B2 receptor tyrosine kinase 2 (*ERBB2*) mutations as they now all have targeted therapies available in the advanced setting.^5^

Targeted therapies have become the standard of care in the adjuvant setting for early-stage NSCLC with common *EGFR* mutations and, most recently, *ALK* fusions. The ADAURA trial showed both overall survival (OS) and disease-free survival (DFS) benefits with the use of the *EGFR* tyrosine kinase inhibitor (TKI) osimertinib as adjuvant treatment post-surgical resection of stage IB-III *EGFR*-mutant NSCLC.^6^ Recently, the ALINA study also showed an improvement in DFS with adjuvant alectinib compared to chemotherapy in patients with resected early-stage *ALK-*positive NSCLC.^7^ However, the efficacy of targeted therapies in NSCLC with other rare driver mutations in the adjuvant setting remains less well-defined. Several clinical trials are recruiting or have been completed to evaluate the benefits of targeted therapies in the perioperative setting for resectable early-stage NSCLC. These include LIBRETTO-432 (NCT04819100), which studies the efficacy of selpercatinib in patients with *RET* fusion early-stage NSCLC post-surgery or radiation, NAUTIKA1 (NCT04302025), a multicenter, phase II study examining neoadjuvant and adjuvant therapies for biomarker-selected patients with *ALK*, *ROS1*, *NTRK*1/2/3 fusions, *BRAF* V600E mutation, *RET* fusions, and *KRAS* G12C expression and GEOMETRY-N (NCT04926831) trial investigating the use of capmatinib pre- and post-surgery in the patients with *MET* exon 14 skipping mutation or high *MET* amplification.^8–10^

With the evolving landscape of adjuvant treatment, there is a need for greater knowledge regarding the outcomes of patients with resected early-stage NSCLC that harbor actionable genomic alterations other than *EGFR* and *ALK*. This knowledge is crucial for optimizing patient outcomes and informing future research efforts to improve the management of early-stage NSCLC. This study aims to explore the survival outcomes of patients with resected early-stage NSCLC that harbor rare targetable driver mutations. Rare targetable driver mutations in our study refer to genomic alterations that occur in less than 15% of NSCLC cases. To our knowledge, this will be among the first few studies exploring this subset of lung cancer in the surgical setting.

## Objectives

This study aims to assess relapse-free survival (RFS) and OS in patients with resected early-stage NSCLC with rare targetable driver mutations, including *EGFR* Exon20 insertion, *KRAS* G12C, *ALK*, *ROS1*, *BRAF*, *NTRK1/2/3*, *MET* exon 14 skipping, *RET*, and *ERBB2* mutations. We also aim to characterize the clinical-demographic profile of patients with early-stage NSCLC harboring these mutations, assess the incidence of brain metastasis, and explore factors that might impact OS and RFS in this study population.

## Methods

This was a retrospective observational study. The study database contains clinical, pathological, and molecular data from patients diagnosed with early-stage NSCLC at institutions within the University Health Network, Toronto, ON, Canada. The database included patients seen in our center from 2015 to 2024.

The study was approved by the Research Ethics Board of the University Health Network with approval number REB#19-5099. All patient data were de-identified to ensure confidentiality.

We included patients with (1) a diagnosis of early-stage NSCLC (stage I–III) based on pathological staging according to the eighth edition of the Cancer Staging Manual of the American Joint Committee on Cancer and Union for International Cancer Control (AJCC); (2) who underwent curative-intent surgery, defined as complete resection of the primary tumor with or without lymph node dissection; and (3) presence of targetable driver mutations, including *EGFR* Exon20 insertion, *KRAS* G12C, *ALK*, *ROS1*, *BRAF*, *MET*, *RET*, and *ERBB2* mutations identified through molecular analysis.

Clinical data extracted from the electronic medical records of eligible patients included age at diagnosis, sex, smoking status (current/former/never-smoker), pathologic stage, cancer histology, type of surgery (lobectomy, pneumonectomy, segmentectomy, or wedge resection), and use of perioperative chemotherapy. Molecular data, including genomic alterations and *TP53* co-mutation, were obtained from pathology reports. We used the STROBE cohort checklist when writing our report.^11^

## Molecular analysis

Molecular analysis of tissue samples was performed as per standard clinical practices at our institution. The molecular analysis methods included validated polymerase chain reaction (PCR)-based assays to detect *EGFR*, break-apart fluorescence in situ hybridization (FISH) and immunohistochemistry (IHC) assays for *ALK* and *ROS1* rearrangements and a targeted next-generation sequencing (NGS) panel for NSCLC. Our study only included genomic alterations classified as Tier I variants of strong clinical significance with evidence of clinical utility that predicts response to targeted therapies.^12^

## Statistical methods

Descriptive statistics were used to summarize the demographic and clinical characteristics of the patients. Categorical variables were reported as frequencies and percentages, while continuous variables were reported as means with standard deviations or medians with ranges. OS is defined as the time from surgical resection to death from any cause. RFS is defined as the time from surgical resection to the first documented relapse of NSCLC or death from any cause, whichever comes first. Kaplan–Meier survival curves estimated OS and RFS probabilities. Associations between baseline characteristics, adjuvant chemotherapy, mutation subtype, TP53 co-mutation, and survival were assessed using Cox regression. The incidence of brain metastases was described with cumulative incidence curves and compared among mutation groups using Gray’s test. OS, RFS, and time to brain metastasis were censored if still alive and at risk after 5 years of follow-up. All statistical analyses were conducted using R statistical software version 4.3.1, with *p*-values < 0.05 considered statistically significant.

## Results

**Figure 1.**
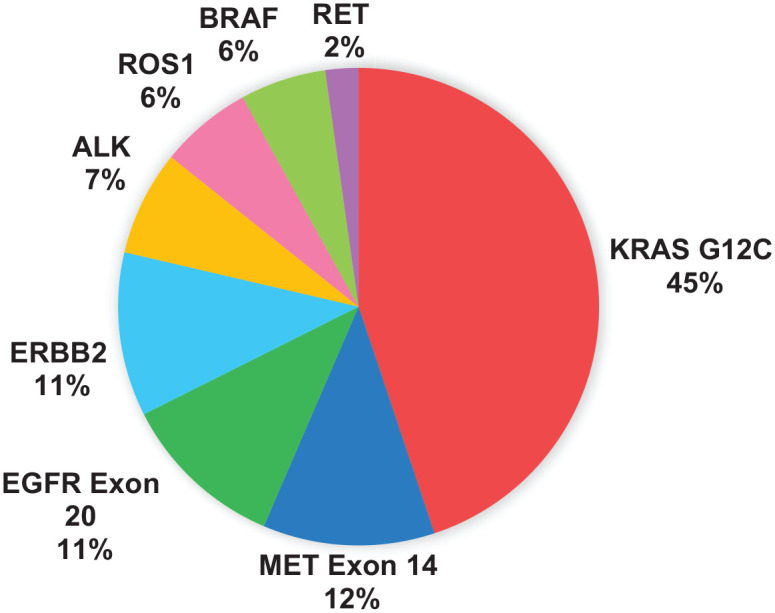
Distribution of patients by mutation subtype.

A total of 225 patients were included in the analysis, and their baseline characteristics are summarized in Table 1. We identified the following mutations (Figure 1): *KRAS* G12C (*n* = 101, 45%), *MET* exon 14 skipping (*n* = 26, 12%), *EGFR* Exon 20 (*n* = 25, 11%), *ERBB2* (*n* = 25, 11%), ALK fusion (*n* = 16, 7%), *ROS1* fusion (*n* = 14, 6%), *BRAF* V600E mutation (*n* = 13, 6%), and *RET* fusion (*n* = 5, 2%) (Table S1). Smoking status was known for 206 patients; only 65 (29%) had no prior history of tobacco use. Smoking was associated with *KRAS* G12C mutation; among 101 patients with *KRAS* G12C mutations, only 3 were never-smokers, compared to 62 of 124 patients (55%) with other mutations (*p* < 0.001).

**Table 1.** Summary of baseline demographics.

Demographic	*N* (%)
Age at diagnosis (years)	
Median (minimum, maximum)	66.5 (40.0, 89.4)
Sex	
Female	139 (62)
Male	86 (38)
Smoking status	
Current or former smoker	141 (63)
Never-smoker	65 (29)
Unknown	19 (8)
Stage at diagnosis	
I	141 (63)
II	43 (19)
III	41 (18)
Histology	
Adenocarcinoma	208 (95)
Mixed	5 (2)
Other	7 (3)
Unknown	5 (2)
Surgery type	
Lobectomy	174 (77)
Wedge resection	33 (15)
Segmentectomy	8 (4)
Pneumonectomy	3 (1)
Other	5 (2)
Unknown	2
Chemotherapy	
Perioperative chemotherapy	70 (32)
Median number of cycles	4
TP53 status	
Wild type	142 (65)
Mutant	75 (35)
Unknown	8

Approximately one-third of patients had TP53 co-mutation (*n* = 75, 35%). TP53 co-mutation was not significantly associated with smoking status (*p* = 0.19). Cancers from most of the never-smokers (72%) were TP53 wild type, although this finding was not statistically significant.

**Figure 2.**
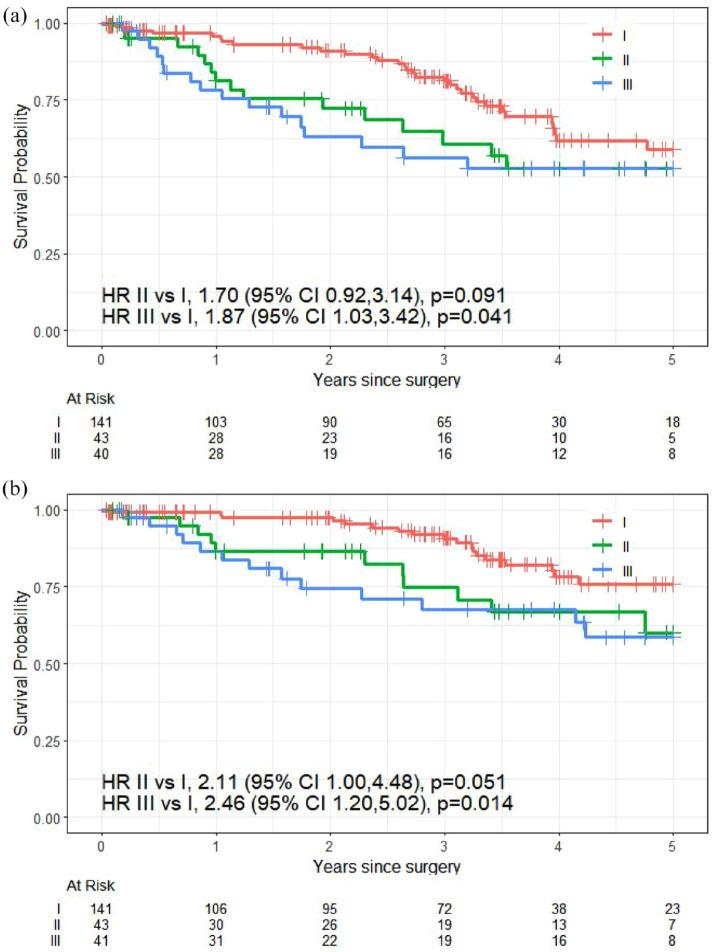
Kaplan–Meier curves of (a) relapse-free survival by stage at diagnosis and (b) overall survival by stage at diagnosis.

The median follow-up for this study was 3.58 years (95% CI: 3.23–3.98). The 5-year RFS probabilities were 59% for stage I (95% CI: 48%, 73%), 53% for stage II (95% CI: 37%, 74%), and 53% for stage III (95% CI: 38%, 73%). The difference in RFS did not reach statistical significance in stage II compared to stage I (HR 1.70, 95% CI: 0.92, 3.14; *p* = 0.091; Figure 2(a)). RFS was significantly shorter in stage III compared to stage I (HR 1.87, 95% CI: 1.03, 3.42; *p* = 0.041).

For all patients, the 5-year survival probabilities were 76% for stage I (95% confidence interval (CI): 66%, 87%), 60% for stage II (95% CI: 43%, 84%), and 58% for stage III (95% CI: 43%, 80%). Compared to stage I patients, OS was significantly shorter in stage II (hazard ratio (HR) 2.11; 95% CI: 1.00, 4.48; *p* = 0.051) and in stage III (HR 2.46; 95% CI: 1.20, 5.02; *p* = 0.014; Figure 2(b)).

**Figure 3.**
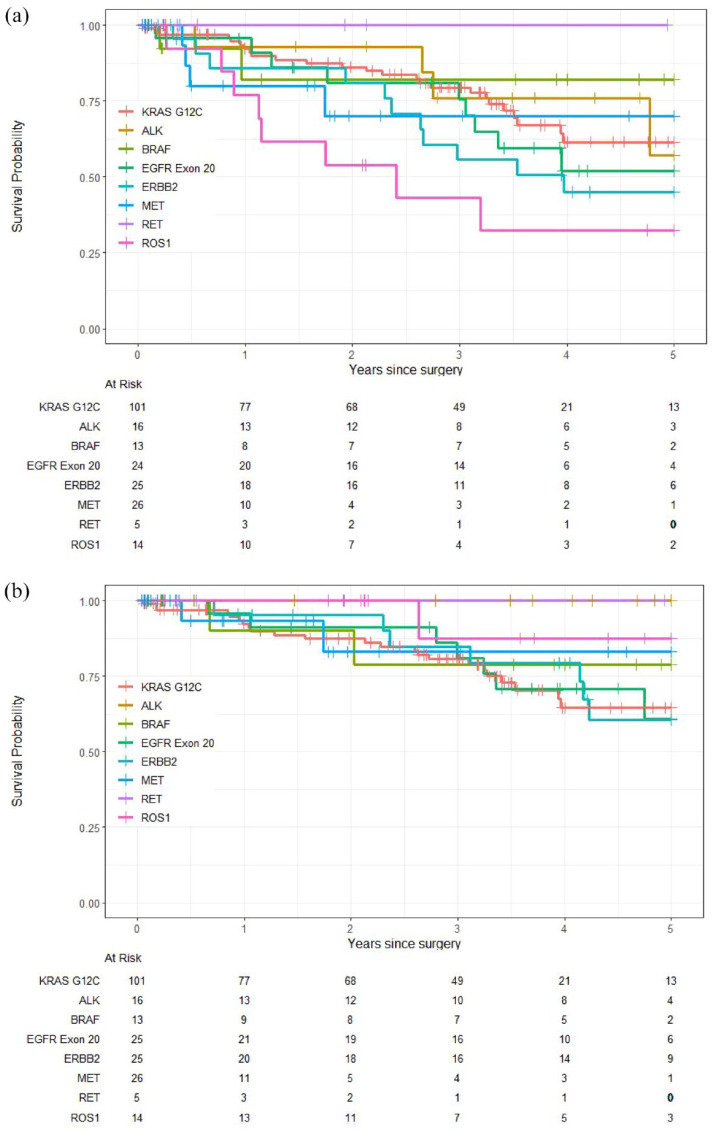
Kaplan–Meier curves of (a) relapse-free survival by mutation subtype and (b) overall survival by mutation subtype.

The analysis of outcomes by mutation (Table 2) showed that most mutation subgroups had numerically shorter RFS than those with *KRAS* G12C except for *BRAF* V600E mutation (HR 0.97, 95% 0.29, 3.24; *p* = 0.96) and RET fusion (no events). The analysis showed that patients with *ROS1* fusion (*n* = 14) had the worst RFS compared to *KRAS* G12C mutation (HR 2.70, 95% CI: 1.18, 6.20; *p* = 0.019). Patients in the pooled fusion mutation subgroup (*ALK*, *ROS1*, and *RET*) showed numerically shorter RFS compared to *KRAS* G12C (HR 1.47, 95% CI: 0.75, 2.88; *p* = 0.26) (Table 2 and Figure 3(a)).

**Table 2.** Summary of the stage-adjusted hazard ratio for RFS and OS by mutation subtype.

Mutation	*N*	RFS			OS		
	225	HR (95% CI)	*p*	5-year survival (95% CI)	HR (95% CI)	*p*	5-year survival (95% CI)
*KRAS* G12C	101	Reference		61% (0.5, 0.76)	Reference		64% (0.53, 0.79)
*ALK*	16	1.05 (0.40, 2.73)	0.93	57% (0.3, 1)	No events	—	100%
*BRAF* V600E	13	0.97 (0.29, 3.24)	0.96	82% (0.62, 1)	0.80 (0.19, 3.45)	0.76	79% (0.56, 1)
*EGFR* exon 20	25	1.41 (0.65, 3.06)	0.38	52% (0.33, 0.82)	0.91 (0.38, 2.16)	0.83	61% (0.4, 0.92)
*ERBB2*	25	1.77 (0.91, 3.46)	0.095	45% (0.27, 0.73)	0.84 (0.36, 1.99)	0.70	60% (0.41, 0.89)
*MET* exon 14	26	1.65 (0.61, 4.47)	0.33	70% (0.49, 1)	0.54 (0.12, 2.40)	0.42	83% (0.63, 1)
*RET*	5	No events	—	100%	No events	—	100%
*ROS1*	14	2.70 (1.18, 6.20)	0.019	32% (0.13, 0.77)	0.22 (0.03, 1.64)	0.14	88% (0.67, 1)
Fusions (*ALK*, *ROS1*, *RET*)	35	1.47 (0.75, 2.88)	0.26	50% (0.32, 0.79)	0.10 (0.01, 0.77)	0.027	95% (0.86, 1)

*BRAF*, B-Raf proto-oncogene; EGFR, epidermal growth factor receptor; *ERBB2*, Erb-B2 receptor tyrosine kinase 2; *MET*, mesenchymal–epithelial transition factor; *NTRK1/2/3*, neurotrophic tyrosine receptor kinase 1/2/3; OS, overall survival; RFS, relapse-free survival; *RET*, rearranged during transfection; *ROS1*, c-ros Oncogene 1.

All mutation subgroups were associated with numerically better OS although the difference was not statistically significant. We also compared the combined fusion mutation subgroup (*ALK*, *ROS1*, and *RET*) to *KRAS* G12C, and identified significantly longer OS compared to *KRAS* G12C (HR 0.10; 95% CI: 0.01, 0.77; *p* = 0.027). Patients with *ALK* and *RET* fusion had 100% 5-year OS as no events were recorded. While the *ROS1* subgroup had the worst RFS, the overall 5-year OS was 88% (95% CI: 0.67, 1) (Table 2 and Figure 3(b)).

At 5 years, patients with *ERBB2* mutations had the highest cumulative incidence of brain metastasis (22%; 95% CI: 6.4%, 44%), followed by those with *EGFR* Exon 20 mutations (19%; 95% CI: 5.7%, 39%). By contrast, *KRAS* G12C mutations had a lower incidence of brain metastasis (0.99%; 95% CI: 0.09%, 4.9%), and those with *MET* exon 14 skipping mutation showed no brain metastasis at 5 years (Table 3).

**Table 3.** Cumulative incidence of brain metastasis at 2 and 5 years by mutation.

Mutation	Year 2 (95% CI)	Year 5 (95% CI)
*KRAS* G12C	0%	0.99% (0.09%, 4.9%)
*EGFR* exon 20	9.2% (1.5%, 26%)	19% (5.7%, 39%)
*ERBB2*	5.3% (0.3%, 22%)	22% (6.4%, 44%)
*MET* exon 14	0%	0%
Other mutations*	7.8% (2.0%, 19%)	19% (5.3%, 39%)
Fusions (*ALK*, *ROS1*, *RET*)	7% (1.2%, 20%)	23% (4.7%, 49%)

* Other mutations include *ALK*, *BRAF* V600E, *RET*, *ROS1*.

*BRAF*, B-Raf proto-oncogene; EGFR, epidermal growth factor receptor; *ERBB2*, Erb-B2 receptor tyrosine kinase 2; *MET*, mesenchymal–epithelial transition factor; *NTRK1/2/3*, neurotrophic tyrosine receptor kinase 1/2/3; *RET*, rearranged during transfection; *ROS1*, c-ros Oncogene 1.

The presence of TP53 co-mutation was associated with a significantly higher risk of death (HR 2.35; 95% CI: 1.26, 4.40; *p* = 0.008) than TP53 wild type.

## Discussion

The identification of multiple mutations in our study, including *KRAS* G12C, *EGFR* Exon 20, *ERBB2*, *MET* exon 14 skipping, *ALK*, *ROS1*, *RET* fusions, and *BRAF* V600E, reflects the molecular heterogeneity of NSCLC. The frequency of these alterations in NSCLC highlights the importance of performing next-generation sequencing (NGS) for full molecular profiling even in early-stage disease to direct potential targeted therapy decision-making after surgical resection. The American Association for Thoracic Surgery (AATS) 2023 Expert Consensus recommends molecular sequencing and biomarker testing for patients with early-stage NSCLC to guide perioperative treatment selection while currently, the NCCN recommends testing only for *EGFR* mutations and *ALK* fusions.^5,13^

Indeed, randomized trials have documented OS and DFS benefits for adjuvant osimertinib and alectinib for *EGFR* and *ALK-*mutated NSCLC, respectively. Osimertinib, an EGFR tyrosine kinase inhibitor (TKI), is now the standard of care for adjuvant treatment for patients with resected, *EGFR*-mutated, stage IB to IIIA NSCLC.^6^ It is currently evaluated in the ADAURA2 trial to assess its efficacy as an adjuvant treatment in patients with resected stage IA2-IA3 *EGFR*-mutated NSCLC.^14^ Alectinib, an ALK TKI, also was recently approved as an adjuvant treatment for patients with completely resected, *ALK*-positive NSCLC of stage IB to IIIA.^7^ With the exception of *KRAS* G12C mutation, however, all other mutation subtypes are much rarer, and although molecularly targeted treatments are available for advanced cancers, less is known about their behavior in the postoperative setting.

In our study, *KRAS G12C* mutation was the most common mutation (45%). We elected to designate *KRAS G12C* as the reference cohort for our study as it is one of the most prevalent mutations in NSCLC, and its prognostic and predictive properties have been well characterized in the adjuvant setting.^15–18^ Five-year RFS and OS results in the current study are similar to those reported by the LACE-Bio group for *KRAS*, thus confirming the interpretation and applicability of our study findings.^17^

In our overall cohort, we observed a higher 5-year OS probability (58%) for stage III resected lung cancer compared to the International Association for the Study of Lung Cancer (IASLC) database, which shows a 5-year survival of 46% for pathological stage IIIA NSCLC.^19^

We observed significant differences in RFS and OS among patients with different mutations. RFS was shorter compared to *KRAS* for most rare subgroups, with a statistically significant difference seen only in the *ROS1* fusion cohort (HR 2.70, *p* = 0.019). However, OS for the *ROS1* fusion cohort was not significantly worse compared to *KRAS* G12C. The fusion mutation subgroup of *ALK*, *ROS1*, and *RET* fusions is associated with better OS but worse RFS than *KRAS* G12C. Thus, our results are similar to previous studies which showed that *ALK* and *ROS1* fusion-positive early-stage NSCLC after curative resection had poorer RFS than *ALK* and *ROS1* fusion-negative NSCLC.^20–22^

Patients with *ERBB2* mutations had the lowest 5-year survival probability at only 60%. However, our findings differed from those of a study on human epidermal growth factor receptor 2 (*HER2*)-mutated lung cancer, which reported a median OS of 89.6 months and a 5-year survival probability of 70% for stage I–III disease.^23^ A meta-analysis of over 6000 patients showed that HER2 protein overexpression is a poor prognostic factor in early-stage NSCLC (HR 1.48 (1.2–0.80), *p* < 0.0001), but only a non-significant trend was seen for *HER2* amplification assessed by FISH (HR 1.14 (0.72–1.83)).^24^ This meta-analysis did not report on *HER2* mutation status.

Despite numerically poorer RFS, however, the OS of all rare mutations showed a trend to be superior to *KRAS* G12C-mutated NSCLC. This dichotomy possibly could be attributed to the use of targeted therapies at relapse. Targeted treatments, such as TKIs for *ALK*, *ROS1*, and *RET* fusions, have shown higher response rates of up to 80% and longer duration of response compared to chemotherapy.^25–27^ KRAS G12C inhibitors have recently been approved for treatment in later-line settings for advanced-stage NSCLC with a response rate of 40%.^28,29^ However, our study did not investigate the association between the use of targeted therapies at relapse and survival outcomes. Further research is needed to validate this potential explanation.

The analysis of brain metastasis showed variability among different types of mutation. Patients with *ERBB2* mutations had the highest cumulative incidence of brain metastasis at 22% at 5 years, while those with *MET* exon 14 skipping mutations did not experience any brain metastasis at 5 years. This finding is similar to a study that showed the cumulative incidence of brain metastasis was higher in *HER2* mutant lung cancer compared to *KRAS* and *EGFR* mutant lung cancer.^30^ This highlights the potential importance of monitoring for central nervous system metastases postoperatively in patients with NSCLC, particularly those with specific mutations.

The presence of TP53 co-mutation was associated with shorter OS. This is consistent with existing literature that TP53 co-mutation is associated with poorer outcomes and could be a prognostic marker in resected early-stage NSCLC.^31,32^

Strengths of this study included its relatively large cohort of patients with rare targetable molecular alterations in a tertiary care clinical setting. Given the limited literature regarding the outcomes of patients with early-stage resected NSCLC with rare mutations, our findings provide valuable real-world evidence regarding their outcomes and prognosis. However, there were several limitations. This was a retrospective single-institution study, which may limit the generalizability of the results; more research is needed to determine whether the same associations persist in different clinical contexts. Although our total sample size is relatively large, the rarity of these molecular alterations limited the statistical power needed to conduct more in-depth multivariable comparisons of some of the mutation subgroups. The study’s short follow-up period also led to immature data regarding OS and RFS. These limitations should be considered when interpreting the results of the study.

## Conclusion

Our study provides an analysis of the outcomes and prognostic factors in patients with resected early-stage NSCLC harboring rare targetable driver mutations. RFS was shorter for most NSCLC with rare targetable mutations compared to *KRAS G12C-*mutated NSCLC. Despite poorer RFS, OS of all mutations was superior to *KRAS G12C*-mutated NSCLC. This suggests a potential role for targeted therapies in the adjuvant setting. These findings could be validated through prospective studies with larger patient cohorts and longer follow-ups. Future research should include clinical trials focusing on adjuvant treatments for rare driver mutations and exploring targeted therapies to improve outcomes for patients with early-stage NSCLC.

## Supplemental Material

sj-docx-1-tam-10.1177_17588359241308466 – Supplemental material for Analysis of outcomes in resected early-stage NSCLC with rare targetable driver mutations Supplemental material, sj-docx-1-tam-10.1177_17588359241308466 for Analysis of outcomes in resected early-stage NSCLC with rare targetable driver mutations by Nadia Ghazali, Jamie Feng, Katrina Hueniken, Khaleeq Khan, Karmugi Balaratnam, Thomas K. Waddell, Kazuhiro Yasufuku, Andrew Pierre, Laura Donahoe, Elliot Wakeam, Marcelo Cypel, Jonathan Yeung, Shaf Keshavjee, Marc de Perrot, Natasha B. Leighl, Geoffrey Liu, Penelope A. Bradbury, Adrian Sacher, Lawson Eng, Tracy Stockley, Ming Sound Tsao and Frances A. Shepherd in Therapeutic Advances in Medical Oncology

sj-docx-2-tam-10.1177_17588359241308466 – Supplemental material for Analysis of outcomes in resected early-stage NSCLC with rare targetable driver mutations Supplemental material, sj-docx-2-tam-10.1177_17588359241308466 for Analysis of outcomes in resected early-stage NSCLC with rare targetable driver mutations by Nadia Ghazali, Jamie Feng, Katrina Hueniken, Khaleeq Khan, Karmugi Balaratnam, Thomas K. Waddell, Kazuhiro Yasufuku, Andrew Pierre, Laura Donahoe, Elliot Wakeam, Marcelo Cypel, Jonathan Yeung, Shaf Keshavjee, Marc de Perrot, Natasha B. Leighl, Geoffrey Liu, Penelope A. Bradbury, Adrian Sacher, Lawson Eng, Tracy Stockley, Ming Sound Tsao and Frances A. Shepherd in Therapeutic Advances in Medical Oncology

## Declarations

**Ethics approval and consent to participate:** We obtained ethics approval from UHN REB. Patient consent was waived due to the retrospective nature of the study.

**Consent for publication:** Yes.

**Availability of data and materials:** The data presented in this study are not publicly available due to the privacy of individuals. The data presented in this study may be made available upon reasonable request from the senior author.

## References

[1] Bray F Laversanne M Sung H , et al. Global cancer statistics 2022: GLOBOCAN estimates of incidence and mortality worldwide for 36 cancers in 185 countries. CA: A Cancer J Clin 2024; 74(3): 229–263. 10.3322/caac.21834 38572751

[2] Datta D Lahiri B. Preoperative evaluation of patients undergoing lung resection surgery. Chest 2003; 123(6): 2096–2103. 12796194 10.1378/chest.123.6.2096

[3] Pignon JP Tribodet H Scagliotti GV , et al. Lung adjuvant cisplatin evaluation: a pooled analysis by the LACE Collaborative Group. J Clin Oncol 2008; 26(21): 3552–3559. 18506026 10.1200/JCO.2007.13.9030

[4] Hirsch FR Suda K Wiens J , et al. New and emerging targeted treatments in advanced non-small-cell lung cancer. Lancet 2016; 388(10048): 1012–1024. 27598681 10.1016/S0140-6736(16)31473-8

[5] National Comprehensive Cancer Network. NCCN clinical practice guidelines in oncology (NCCN Guidelines®) for non-small cell lung cancer V.8.2024. National Comprehensive Cancer Network, 2024. 10.6004/jnccn.2204.0023 38754467

[6] Tsuboi M Herbst RS John T , et al. Overall survival with osimertinib in resected EGFR-mutated NSCLC. N Engl J Med 2023; 389(2): 137–147. 37272535 10.1056/NEJMoa2304594

[7] Wu Y-L Dziadziuszko R Ahn Jin S , et al. Alectinib in resected ALK-positive non–small-cell lung cancer. New England J Med 2024; 390(14): 1265–1276. 38598794 10.1056/NEJMoa2310532

[8] Tsuboi M Goldman JW Wu Y-L , et al. LIBRETTO-432, a phase III study of adjuvant selpercatinib or placebo in stage IB-IIIA RET fusion-positive non-small-cell lung cancer. Future Oncol 2022; 18(28): 3133–3141. 35950566 10.2217/fon-2022-0656

[bibr9-17588359241308466] Lee JM Awad MM Saliba TR , et al. Neoadjuvant and adjuvant capmatinib in resectable non–small cell lung cancer with MET exon 14 skipping mutation or high MET amplification: GEOMETRY-N trial. J Clin Oncol 2022; 40(16_suppl): TPS8590-TPS.

[10] Lee JM Toloza EM Pass HI , et al. P2.01-06 NAUTIKA1 study: preliminary efficacy and safety data with neoadjuvant alectinib in patients with stage IB-III ALK+ NSCLC. J Thoracic Oncol 2023; 18(11): S297–S8.

[11] von Elm E Altman DG Egger M , et al. The strengthening the reporting of observational studies in epidemiology (STROBE) statement: guidelines for reporting observational studies. Int J Surg 2014; 12(12): 1495–1499. 25046131 10.1016/j.ijsu.2014.07.013

[12] Li MM Datto M Duncavage EJ , et al. Standards and guidelines for the interpretation and reporting of sequence variants in cancer: a Joint Consensus Recommendation of the Association for Molecular Pathology, American Society of Clinical Oncology, and College of American Pathologists. J Mol Diagn 2017; 19: 4–13. 27993330 10.1016/j.jmoldx.2016.10.002 PMC5707196

[13] Kidane B Bott M Spicer J , et al. The American Association for Thoracic Surgery (AATS) 2023 Expert Consensus Document: Staging and multidisciplinary management of patients with early-stage non-small cell lung cancer. J Thorac Cardiovasc Surg 2023; 166(3): 637–654. 37306641 10.1016/j.jtcvs.2023.04.039

[14] Tsutani Y Goldman JW Dacic S , et al. Adjuvant osimertinib vs. placebo in completely resected stage IA2-IA3 EGFR-mutated NSCLC: ADAURA2. Clin Lung Cancer 2023; 24(4): 376–380. 36872181 10.1016/j.cllc.2023.02.002

[15] Fung AS Karimi M Michiels S , et al. Prognostic and predictive effect of KRAS gene copy number and mutation status in early stage non-small cell lung cancer patients. Transl Lung Cancer Res 2021; 10(2): 826–838. 33718025 10.21037/tlcr-20-927 PMC7947394

[bibr16-17588359241308466] Martin P Leighl NB Tsao MS , et al. KRAS mutations as prognostic and predictive markers in non-small cell lung cancer. J Thorac Oncol 2013; 8(5): 530–542. 23524403 10.1097/JTO.0b013e318283d958

[bibr17-17588359241308466] Shepherd FA Domerg C Hainaut P , et al. Pooled analysis of the prognostic and predictive effects of KRAS mutation status and KRAS mutation subtype in early-stage resected non-small-cell lung cancer in four trials of adjuvant chemotherapy. J Clin Oncol 2013; 31(17): 2173–2181. 23630215 10.1200/JCO.2012.48.1390 PMC4881333

[18] Shepherd FA Lacas B Le Teuff G , et al. Pooled analysis of the prognostic and predictive effects of TP53 comutation status combined with KRAS or EGFR mutation in early-stage resected non-small-cell lung cancer in four trials of adjuvant chemotherapy. J Clin Oncol 2017; 35(18): 2018–2027. 28453411 10.1200/JCO.2016.71.2893 PMC6075828

[19] Goldstraw P Chansky K Crowley J , et al. The IASLC lung cancer staging project: proposals for revision of the TNM stage groupings in the forthcoming (Eighth) edition of the TNM classification for lung cancer. J Thorac Oncol 2016; 11(1): 39–51. 26762738 10.1016/j.jtho.2015.09.009

[20] Chaft JE Dagogo-Jack I Santini FC , et al. Clinical outcomes of patients with resected, early-stage ALK-positive lung cancer. Lung Cancer 2018; 122: 67–71. 30032847 10.1016/j.lungcan.2018.05.020 PMC6062851

[bibr21-17588359241308466] Yang P Kulig K Boland JM , et al. Worse disease-free survival in never-smokers with ALK+ lung adenocarcinoma. J Thorac Oncol 2012; 7(1): 90–97. 22134072 10.1097/JTO.0b013e31823c5c32 PMC3931519

[22] Kim MH Shim HS Kang DR , et al. Clinical and prognostic implications of ALK and ROS1 rearrangements in never-smokers with surgically resected lung adenocarcinoma. Lung Cancer 2014; 83(3): 389–395. 24462463 10.1016/j.lungcan.2014.01.003

[23] Mazières J Peters S Lepage B , et al. Lung cancer that harbors an HER2 mutation: epidemiologic characteristics and therapeutic perspectives. J Clin Oncol 2013; 31(16): 1997–2003. 23610105 10.1200/JCO.2012.45.6095

[24] Liu L Shao X Gao W , et al. The role of human epidermal growth factor receptor 2 as a prognostic factor in lung cancer: a meta-analysis of published data. J Thorac Oncol 2010; 5(12): 1922–1932. 21155183 10.1097/jto.0b013e3181f26266

[25] Solomon BJ Kim D-W Wu Y-L , et al. Final overall survival analysis from a study comparing first-line crizotinib versus chemotherapy in ALK-mutation-positive non–small-cell lung cancer. J Clin Oncol 2018; 36(22): 2251–2258. 29768118 10.1200/JCO.2017.77.4794

[bibr26-17588359241308466] Shaw AT Ou S-HI Bang Y-J , et al. Crizotinib in ROS1-rearranged non-small-cell lung cancer. N Engl J Med 2014; 371: 1963–1971. 25264305 10.1056/NEJMoa1406766 PMC4264527

[27] Zhou C Solomon B Loong Herbert H , et al. First-line selpercatinib or chemotherapy and pembrolizumab in RET fusion–positive NSCLC. New England J Med 2023; 389(20): 1839–1850. 37870973 10.1056/NEJMoa2309457 PMC10698285

[28] de Langen AJ Johnson ML Mazieres J , et al. Sotorasib versus docetaxel for previously treated non-small-cell lung cancer with KRAS^G12C^ mutation: a randomised, open-label, phase 3 trial. Lancet 2023; 401(10378): 733–746. 36764316 10.1016/S0140-6736(23)00221-0

[29] Jänne PA Riely GJ Gadgeel SM , et al. Adagrasib in non-small-cell lung cancer harboring a KRAS(G12C) mutation. N Engl J Med 2022; 387: 120–131. 35658005 10.1056/NEJMoa2204619

[30] Offin M Feldman D Ni A , et al. Frequency and outcomes of brain metastases in patients with HER2-mutant lung cancers. Cancer 2019; 125(24): 4380–4387. 31469421 10.1002/cncr.32461 PMC6891113

[31] Labbé C Cabanero M Korpanty GJ , et al. Prognostic and predictive effects of TP53 co-mutation in patients with EGFR-mutated non-small cell lung cancer (NSCLC). Lung Cancer 2017; 111: 23–29. 28838393 10.1016/j.lungcan.2017.06.014

[32] Ma X Le Teuff G Lacas B , et al. Prognostic and predictive effect of TP53 mutations in patients with non-small cell lung cancer from adjuvant cisplatin-based therapy randomized trials: a LACE-bio pooled analysis. J Thorac Oncol 2016; 11(6): 850–861. 26899019 10.1016/j.jtho.2016.02.002

