# Supplementary material for: Analysis of outcomes in resected early-stage NSCLC with rare targetable driver mutations
Source: Ther Adv Med Oncol. 2024 Dec 23;16:17588359241308466. doi: 10.1177/17588359241308466 (PMC11672496; doi:10.1177/17588359241308466)
Supplement: sj-docx-1-tam-10.1177_17588359241308466 – Supplemental material for Analysis of outcomes in resected early-stage NSCLC with rare targetable driver mutations [file sj-docx-1-tam-10.1177_17588359241308466.docx]

Supplemental material

Table.S1: Summary of baseline demographics based on each mutation

|  | **Full Sample**  **N (%)** | **ALK**  **N (%)** | **BRAF V600E**  **N (%)** | **EGFR Exon 20**  **N (%)** | **ERBB2**  **N (%)** | **KRAS G12C**  **N (%)** | **MET**  **N (%)** | **RET**  **N (%)** | **ROS1**  **N (%)** |
| --- | --- | --- | --- | --- | --- | --- | --- | --- | --- |
| **Number** | 225 | 16 | 13 | 25 | 25 | 101 | 26 | 5 | 14 |
| **Age at diagnosis** |  |  |  |  |  |  |  |  |  |
| Median (Min,Max) | 66.5 (40.0, 89.4) | 60.5 (42.1, 75.0) | 66.4 (41.3, 82.8) | 64.8 (45.4, 84.8) | 65.2 (44.7, 81.8) | 68.2 (50.6, 84.2) | 75.8 (48.5, 89.4) | 64.4 (54.1, 76.1) | 59.3 (40.0, 74.4) |
| **Sex** |  |  |  |  |  |  |  |  |  |
| Female | 139 (62) | 12 (75) | 8 (62) | 17 (68) | 13 (52) | 58 (57) | 18 (69) | 3 (60) | 10 (71) |
| Male | 86 (38) | 4 (25) | 5 (38) | 8 (32) | 12 (48) | 43 (43) | 8 (31) | 2 (40) | 4 (29) |
| **Smoking status** |  |  |  |  |  |  |  |  |  |
| Current or former smoker | 141 (63) | 4 (25) | 6 (47) | 12 (48) | 7 (28) | 91 (90) | 15 (58) | 1 (20) | 5 (36) |
| Never smoker | 65 (29) | 10 (62) | 6 (46) | 11 (44) | 16 (64) | 3 (3) | 8 (31) | 3 (60) | 8 (57) |
| Unknown | 19 (8) | 2 (12) | 1 (8) | 2 (8) | 2 (8) | 7 (7) | 3 (12) | 1 (20) | 1 (7) |
| **Stage at diagnosis** |  |  |  |  |  |  |  |  |  |
| I | 141 (63) | 10 (62) | 10 (77) | 14 (56) | 13 (52) | 66 (65) | 18 (69) | 3 (60) | 7 (50) |
| II | 43 (19) | 3 (19) | 3 (23) | 3 (12) | 5 (20) | 21 (21) | 3 (12) | 2 (40) | 3 (21) |
| III | 41 (18) | 3 (19) | 0 (0) | 8 (32) | 7 (28) | 14 (14) | 5 (19) | 0 (0) | 4 (29) |
| **Histology** |  |  |  |  |  |  |  |  |  |
| Adenocarcinoma | 208 (95) | 16 (100) | 13 (100) | 22 (88) | 24 (96) | 91 (95) | 23 (88) | 5 (100) | 14 (100) |
| Mixed | 5 (2) | 0 (0) | 0 (0) | 2 (8) | 1 (4) | 0 (0) | 2 (8) | 0 (0) | 0 (0) |
| Other | 7 (3) | 0 (0) | 0 (0) | 1 (4) | 0 (0) | 5 (5) | 1 (4) | 0 (0) | 0 (0) |
| Unknown | 5 | 0 | 0 | 0 | 0 | 5 | 0 | 0 | 0 |
| **Surgery type** |  |  |  |  |  |  |  |  |  |
| Lobectomy | 174 (77) | 15 (94) | 9 (69) | 23 (92) | 21 (84) | 73 (73) | 18 (70) | 5 (70) | 10 (77) |
| Wedge resection | 33 (15) | 0 (0) | 2 (15) | 1 (4) | 3 (12) | 20 (20) | 5 (19) | 0 (0) | 2 (16) |
| Segmentectomy | 8 (4) | 1 (6) | 1 (8) | 0 (0) | 0 (0) | 3 (3) | 3 (12) | 0 (0) | 0 (0) |
| Pneumonectomy | 3 (1) | 0 (0) | 0 (0) | 0 (0) | 1 (4) | 1 (1) | 0 (0) | 0 (0) | 1 (8) |
| Other | 5 (2) | 0 (0) | 1 (8) | 1 (4) | 0 (0) | 3 (3) | 0 (0) | 0 (0) | 0 (0) |
| Unknown | 2 | 0 | 0 | 0 | 0 | 1 | 0 | 0 | 1 |
| **TP53 status** |  |  |  |  |  |  |  |  |  |
| Wild-type | 142 (65) | 15 (94) | 7 (58) | 13 (57) | 16 (67) | 60 (61) | 17 (68) | 5 (100) | 9 (64) |
| Mutant | 75 (35) | 1 (6) | 5 (42) | 10 (43) | 8 (33) | 38 (39) | 8 (32) | 0 (0) | 5 (36) |
| Unknown | 8 | 0 | 1 | 2 | 1 | 3 | 1 | 0 | 0 |
